# High Osmotic Stress Increases OmpK36 Expression through the Regulation of KbvR to Decrease the Antimicrobial Resistance of Klebsiella pneumoniae

**DOI:** 10.1128/spectrum.00507-22

**Published:** 2022-06-06

**Authors:** Meng Wang, Yujiao Tian, Li Xu, Fusheng Zhang, Huigai Lu, Moran Li, Bei Li

**Affiliations:** a School of Basic Medicine, Hubei University of Medicine, Shiyan, Hubei, People’s Republic of China; b Biomedical Research Institute, Hubei University of Medicine, Shiyan, Hubei, People’s Republic of China; c Hubei Clinical Research Center for Precise Diagnosis and Treatment of Liver Cancer, Shiyan, Hubei, People’s Republic of China; University of Manitoba

**Keywords:** *Klebsiella pneumoniae*, osmotic stress, antimicrobial resistance, OmpK36, KbvR

## Abstract

Klebsiella pneumoniae is a pathogen known for its high frequency of antimicrobial resistance. Responses to various environmental stresses during its life can influence the resistance to antibiotics. Here, we demonstrate the role and mechanism of KbvR regulator in the response to environmental osmotic stress and in the effect of osmotic stress on antimicrobial resistance. The *kbvR* mutant strain exhibited increasing tolerance to high osmotic stress and certain antibiotics, including β-lactams. The expression levels of KbvR and outer membrane porin OmpK36 were upregulated in response to high osmotic stress in the wild type (WT), and the deletion of *kbvR* decreased the expression level of *ompK36*. The membrane permeability of the *kbvR* mutant strain was decreased, which was partly restored through the upregulated expression of OmpK36. The DNA affinity purification sequencing (DAP-seq) and microscale thermophoresis (MST) assay disclosed the binding of KbvR to the promoter of the *ompK36* gene, indicating that KbvR directly and positively regulated the expression of OmpK36. The high osmotic stress increased the susceptibility to β-lactams and the expression of *ompK36* in the WT strain. However, the increased *ompK36* expression and the susceptibility to β-lactams in the *kbvR* mutant strain under high osmotic stress were lower than those of WT. In conclusion, our study has identified that high osmotic stress in the environment influenced the resistance of K. pneumoniae to antibiotics and that the regulation of KbvR with OmpR on the expression of OmpK36 was involved in countering high osmotic stress to change the antimicrobial resistance.

**IMPORTANCE** Klebsiella pneumoniae is considered a global threat because of the rising prevalence of multidrug-resistant strains and their optimal adaptation to clinical environments and the human host. The sensing and adaption abilities of bacteria to the environmental osmotic stress can change the expression of their outer membrane porins, membrane permeability, and resistance to antibiotics. This study reports that KbvR is a newly found regulator that can be upregulated under high osmotic stress and directly regulate the expression of OmpK36 to change the resistance of K. pneumoniae to β-lactam antibiotics. The results demonstrate how adaptation to high osmotic stress changes the sensitivity of K. pneumoniae to antibiotics. The mechanism can be used to sensitize bacteria to antibiotics and highlight new potential strategies for exploiting shared constraints in governing adaptation to diverse environmental challenges.

## INTRODUCTION

Klebsiella pneumoniae is an opportunistic pathogen that causes a wide range of nosocomial infections, including pneumonia, urinary tract infection, and soft tissue infection (1, 2). Few hypervirulent variants with unique hypermucoviscous (hypervirulent) phenotype can cause serious community-acquired invasive infections, such as pyogenic liver abscess, endophthalmitis, and meningitis (3–5). Additionally, K. pneumoniae often displays multidrug-resistance phenotypes that make it difficult to choose appropriate antibiotics for treatment (6–8). In nature, K. pneumoniae could be widely found in the mouth, skin, and intestine of mammals and in environmental sources, such as soils, water surfaces, and medical devices (9). In these environments, K. pneumoniae encounters different stresses associated with variations in pH, oxidative stress, and osmotic stress (10). Pathogens adopt different mechanisms to respond to these stresses (11, 12), which might link to antimicrobial resistance (13–15).

Osmolarity is a fundamental environmental stress that ranges from low osmotic stress, such as that in fresh water, to high osmotic stress, such as that in soil and human intestine. A previous study found that high-osmolarity medium could increase the transcriptional level of OmpC (OmpK36) in Escherichia coli (16). OmpK36 (OmpC) and OmpK35 (OmpF) are the major abundant proteins in the outer membrane of K. pneumoniae, and they form hydrophilic channels (porins) to facilitate the passage of nutrients and certain antibiotics through the outer membrane (17). The absence or downregulation of OmpK35 and OmpK36 has been implicated for resistance to a wide range of antibiotics, including β-lactams (18–20). The high osmolarity of the surrounding medium can be sensed by the two-component system EnvZ–OmpR to alter the transcriptional level of *ompC*, which partly explains the increase in antimicrobial susceptibility (16, 21). Except for OmpR, the biosynthesis of OmpK36 (OmpC) in K. pneumoniae might be regulated by KbvR, a LuxR-type regulator whose location is predicted in cytoplasm by ProtComp Version 9 software. In our previous research, we found that this regulator contributes to the pathogenicity of K. pneumoniae by regulating antiphagocytosis abilities, capsule production, and biofilm formation. The transcriptome sequencing (RNA-seq) results showed that the expression levels of outer membrane proteins such as OmpK36, OmpA, and OmpW were decreased in the *kbvR* mutant strain (22). The role and mechanism of KbvR in the resistance to osmotic pressure and antibiotics need to be studied further.

In this study, we investigated the role and mechanism of KbvR in response to high osmotic stress and evaluated the influence of high osmotic stress on antibiotics resistance. Results show that KbvR negatively regulates the responses to high osmotic stress and that high osmotic stress influences the susceptibility of K. pneumoniae to multiple drugs. Furthermore, this work highlights the identification of OmpK36 as a directly regulated protein by KbvR and its role in regulating membrane permeability during high osmotic stress to influence the resistance to antibiotics.

## RESULTS AND DISCUSSION

### Effect of NaCl stress on the survival rate of K. pneumoniae and the expression of *kbvR*.

**FIG 1:**
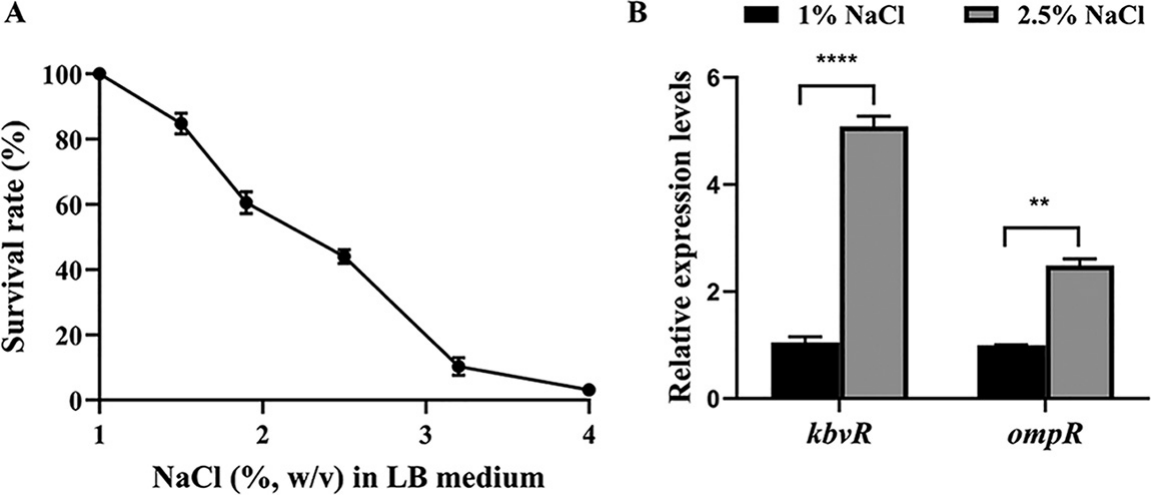
Effect of sodium chloride (NaCl) stress on the survival rate of K. pneumoniae and the expression of *kbvR* and *ompR*. (A) The survival rate of NTUH-K2044 WT strain in different osmotic stress levels of NaCl. The WT strain was grown in LB medium with 1%, 1.5%, 1.9%, 2.5%, 3.2%, or 4% NaCl at 37°C for 3 h with shaking, and CFU were measured by serial dilution and plating. Survival rate was calculated by dividing the CFU of the samples treated with different concentrations of NaCl by the corresponding CFU of the samples with 1% NaCl and multiplying by 100. (B) Effect of NaCl stress on *kbvR* and *ompR* transcription in the WT strain. The transcript levels of *kbvR* and *ompR* were determined by qRT-PCR for the WT strain cultured in the LB broth with 1% or 2.5% NaCl for 3 h. Data are presented as mean ± standard error of the mean (SEM) and representative of three independent experiments. **, *P < *0.01; ****, *P < *0.001 by Student’s *t* test.

NaCl stress is a common osmotic stress encountered in the environmental and intestinal phases of K. pneumoniae. Pathogens respond to this osmotic stress to survive and cause infection. The survival rates of WT strain grown in LB broth with different concentrations of NaCl were calculated to determine the tolerance of K. pneumoniae to the NaCl stress. The addition of NaCl at the concentrations of 1.9%, 2.5%, and 3.2% in LB medium (these salt concentrations are considered to have an osmolarity that nearly mimics that of the intestinal lumen or is even greater [23]) decreased the survival rate to about 67%, 42%, and 10% compared with the growth of the NTUH-K2044 strain under physiological osmotic stress (1% NaCl in LB medium). When higher osmotic stress (4% NaCl) was used, only 1% of bacteria survived (Fig. 1A). Quantitative real-time PCR (qRT-PCR) analysis of *kbvR* was performed at 2.5% NaCl to determine if *kbvR* expression was modified in response to NaCl stress. The expression of KbvR was substantially induced by 5-fold (Fig. 1B). The expression of OmpR, a known regulator that senses osmolarity of the surrounding medium, was also increased (24, 25). These results suggest that K. pneumoniae has certain resistance to environmental osmotic stress, and KbvR might take part in this adaption.

### Deletion of *kbvR* increases the tolerance of K. pneumoniae to osmotic stress and certain antibiotics.

**FIG 2:**
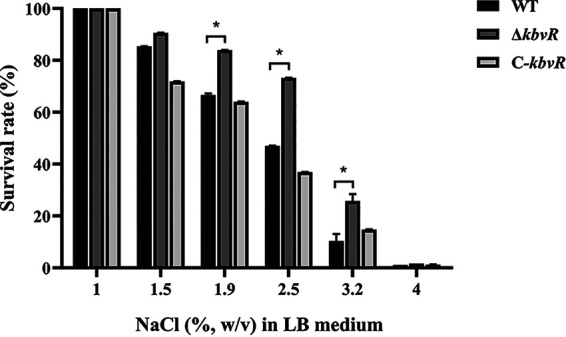
Sensitivity of WT, Δ*kbvR*, and C*-kbvR* strains to osmotic stress. The overnight cultured WT, Δ*kbvR*, and C*-kbvR* strains were diluted 100-fold in fresh LB broth and incubated at 37°C to an OD_600_ of 1.6. Bacteria at 10^7^ CFU mL^−1^ were cultured in 3 mL of LB broth with 1%, 1.5%, 1.9%, 2.5%, 3.2%, or 4% NaCl and incubated at 37°C for 3 h. Survival rate was calculated by dividing the CFU of the samples treated with different concentrations of NaCl by the corresponding CFU of the samples with 1% NaCl and multiplying by 100. Data are presented as mean ± SEM and representative of three independent experiments. *, *P < *0.05 by Student’s *t* test.

The *kbvR* gene was upregulated when the NTUH-K2044 strain was grown in LB medium with 2.5% NaCl supplementation. Hence, the KbvR regulator may play a role in osmotic stress response. As such, we investigated the survival rate of WT, Δ*kbvR*, and C*-kbvR* strains by using LB medium with NaCl at concentrations of 1%, 1.5%, 1.9%, 2.5%, 3.2%, and 4%. After 3 h of growth, no difference was found between the growth of the WT strain and that of the *kbvR* mutant strain in LB medium with 1% NaCl (physiological osmotic stress) (22). Meanwhile, the *kbvR* mutant strain showed increased viability compared to that of the WT strain at 1.9% (83.93% versus 66.67%, *P < *0.05), 2.5% (73.21% versus 43.03%, *P < *0.05), and 3.2% (25.72% versus 10.30%, *P < *0.05) NaCl (Fig. 2). The C-*kbvR* strain exhibited an ability to tolerate osmotic stress similar to that of the WT strain. These results show that KbvR negatively regulates the tolerance to osmotic stress.

The effect of KbvR regulator on antimicrobial resistance was determined by broth microdilution test. The deletion of *kbvR* increased the MIC of K. pneumoniae as follows: 4-fold increases in cefoxitin, polymyxin B, and chloramphenicol MICs and 8-fold increase in cefazolin MIC (Table 1). The increased MICs could be partly restored to the original levels following *kbvR* complementation. The results show that the *kbvR* mutant has additional resistance to β-lactam antibiotics, namely, cefazolin and cefoxitin. K. pneumoniae is one of the bacterial pathogens known for its high frequency of β-lactam antimicrobial resistance, a characteristic that is often associated with reduced outer membrane permeability. OmpK35 and OmpK36 are the major porins in the outer membrane of K. pneumoniae and are shown to be relevant for penetration of β-lactams. β-Lactam antibiotics pass the outer membrane through these porins to access their targets. The lack of production of either or both of these porins can increase the MICs of certain β-lactams (18, 19, 26). The RNA-seq results of the WT strain and the Δ*kbvR* strain showed that the expression level of OmpK36 was obviously decreased in the latter (22). Hence, the downregulation of OmpK36 expression could be responsible for the alteration of membrane permeability and the antimicrobial resistance in the *kbvR* mutant.

**TABLE 1:** MICs of antibiotics for K. pneumoniae WT, Δ*kbvR*, and C-*kbvR* strains

	MIC (μg mL^−1^)			Fold changes in MICs
Antimicrobial agent	WT	Δ*kbvR*	C-*kbvR*	Δ*kbvR*/WT
Gentamicin	4	4	4	1
Streptomycin	16	16	16	1
Ceftazidime	0.5	0.5	0.5	1
Cefoxitin	4	16	8	4
Cefazolin	4	32	8	8
Imipenem	128	128	32	1
Vancomycin	>2048	>2048	>2048	1
Erythromycin	512	512	512	1
Polymyxin B	4	16	8	4
Tetracycline	8	8	8	1
Chloramphenicol	16	64	16	4
Ciprofloxacin	<0.25	<0.25	<0.25	1
Ertapenem	0.03125	0.03125	0.015625	1

### OmpK36 regulated by KbvR plays a role in resistance to antibiotics.

**FIG 3:**
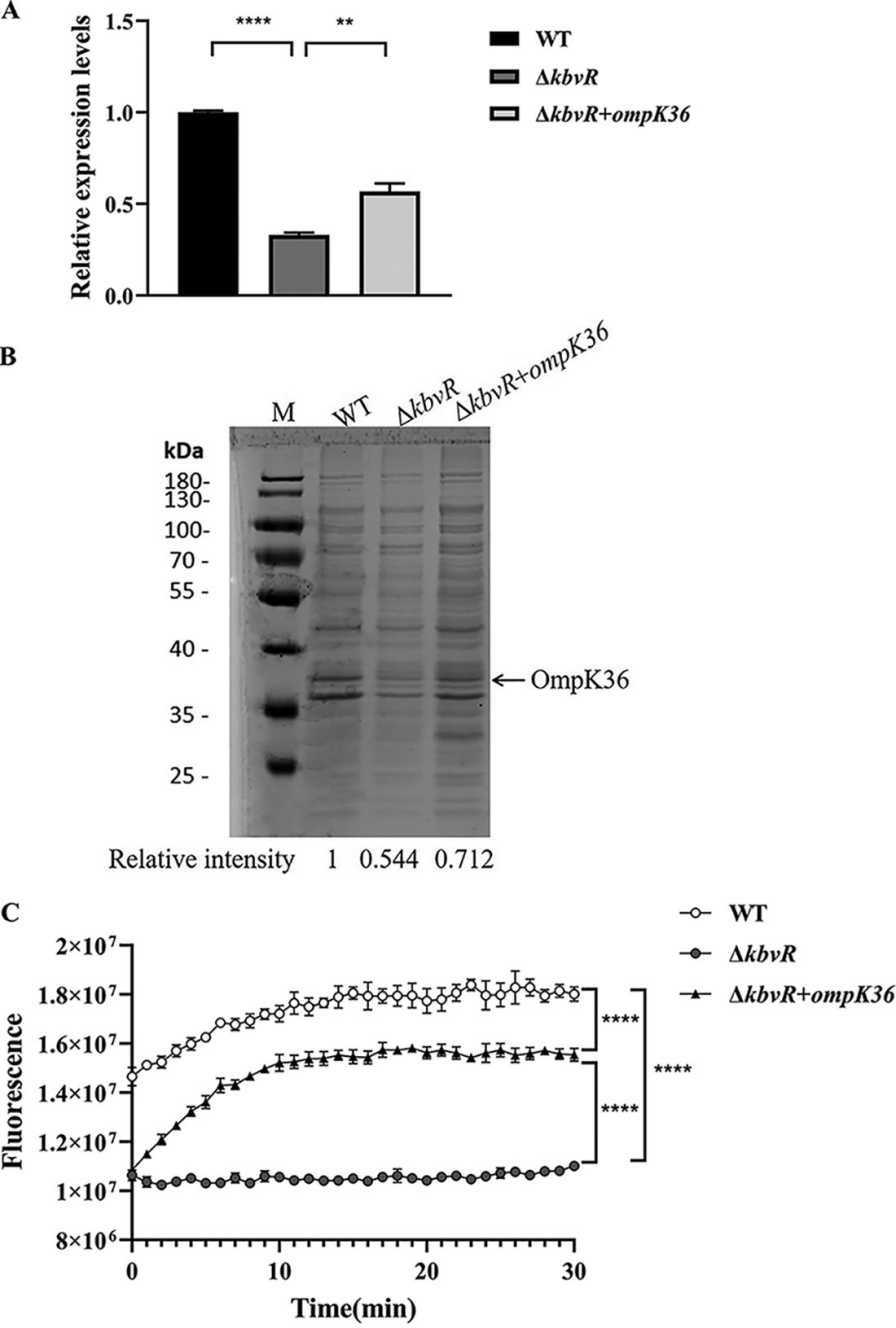
Regulation of KbvR on OmpK36. (A) qRT-PCR. The mRNA levels of *ompK36* in WT, Δ*kbvR*, and Δ*kbvR *+ *ompK36* strains cultured in LB broth with 1% NaCl were compared. The 16S rRNA was amplified as the control. **, *P < *0.01; ****, *P < *0.0001 by Student’s *t* test. (B) SDS-PAGE analysis. The protein levels of OmpK36 in WT, Δ*kbvR*, and Δ*kbvR *+ *ompK36* strains cultured in LB broth were compared by SDS-PAGE analysis. The position of OmpK36 on the gel is indicated by an arrow. The relative intensity of OmpK36 was quantitated by densitometry using ImageJ software. It was calculated by dividing the densitometry of OmpK36 band by the corresponding densitometry of total protein with the protein level of OmpK36 in WT defined as 1. (C) *N*-phenyl-1-naphthylamine (NPN) uptake assay to measure outer membrane permeability. WT, Δ*kbvR*, and Δ*kbvR + ompK36* strains were grown to an OD_600_ of 1.6 in LB broth with 1% NaCl and then analyzed for uptake of NPN in the presence of CCCP. Fluorescence signal was measured within 2 min every 1 min for 30 min. ****, *P < *0.0001 by two-way ANOVA.

The *ompK36* expression in WT and Δ*kbvR* strains was determined by qRT-PCR and outer membrane (OM) permeability assay to confirm if KbvR positively regulates the expression of OmpK36. The qRT-PCR and SDS-PAGE showed a decrease in the *ompK36* expression in the Δ*kbvR* strain, similar to the result of RNA-seq (Fig. 3A and B). The Δ*kbvR* substantially had about 67% lower fluorescence than WT in the N-phenyl-1-naphthylamine (NPN) uptake assay (Fig. 3C). This finding was correlated with decreased OmpK36 porin production in the Δ*kbvR* strain. We hypothesized that the regulation of OmpK36 expression by KbvR plays a role in changing OM permeability and confers multidrug resistance to the *kbvR* mutant strain. To address this problem, we cloned *ompK36* into km-pGEM-T-easy and transformed it into the *kbvR* mutant to obtain the upexpression strain Δ*kbvR* *+* *ompK36* and determine the multidrug resistance phenotype conferred by OmpK36 in K. pneumoniae strain harboring deletion in *kbvR*. The amount of fluorescence of Δ*kbvR *+ *ompK3*6 in the presence of NPN was higher than that of Δ*kbvR* but lower than that of the WT strain, indicating that the regulation of KbvR on the expression of OmpK36 plays a role in the effect of OM permeability. The reasons for not fully restored OM permeability in the Δ*kbvR *+ *ompK3*6 strain were deduced as follows. (i) The expression of OmpK36 in the Δ*kbvR *+ *ompK3*6 strain was upregulated compared with that in the Δ*kbvR* strain but was not fully restored to the expression level in the WT strain (Fig. 3A and B). The km-pGEM-T-easy plasmid used to express OmpK36 is not a continuous expression plasmid, while the expression of OmpK36 is under the control of the regulators of OmpK36. (ii) The upexpression of OmpK36 alone could restore, though not sufficiently, the outer membrane permeability in the *kbvR* mutant to allow the uptake of the fluorescent dye (Fig. 3C). In addition to OmpK36, the RNA-seq showed that some other outer membrane proteins and efflux pump proteins, including OmpA, OmpX, and AcrAB-TolC, were regulated by KbvR, which also influenced the outer membrane permeability (22, 27, 28).

The increased expression of OmpK36 in the Δ*kbvR *+ *ompK3*6 strain restored the susceptibility to β-lactams with decreasing cefazolin MIC (8-fold) and cefoxitin MIC (2-fold) relative to the MICs of Δ*kbvR* (Table 2). Hence, KbvR influences the resistance to β-lactams mainly through the regulation of OmpK36. However, when the expression of OmpK36 was increased in Δ*kbvR *+ *ompK3*6, the susceptibility to polymyxin B was not different from that in the Δ*kbvR* strain, implying that the resistant phenotype to polymyxin B of Δ*kbvR* is not dependent on the regulation of OmpK36. Polymyxin B is a hydrophobic antibiotic that binds to the lipid A of lipopolysaccharide (LPS), weakens the outer membrane barrier, and penetrates by a self-promoted pathway, not by porins (29). The MICs of chloramphenicol decreased by 2-fold in Δ*kbvR + ompK36* compared to those in WT. This finding may be attributed to the downregulation of KbvR on the expression of AcrAB-TolC efflux pump, which influences the susceptibility to chloramphenicol with the outer membrane porins by decreasing the drug efflux from the cytoplasm (30–32).

**TABLE 2:** MICs of antibiotics for K. pneumoniae WT, Δ*kbvR*, and Δ*kbvR *+ *ompK36* strains

	MIC (μg mL^−1^)			Fold changes in MICs
Antimicrobial agent	WT	Δ*kbvR*	Δ*kbvR + ompK36*	Δ*kbvR/*Δ*kbvR + ompK36*
Cefoxitin	4	16	8	2
Cefazolin	4	32	4	8
Polymyxin B	4	16	16	1
Chloramphenicol	16	64	8	8

### KbvR directly regulates the expression of OmpK36.

The RNA-seq and qRT-PCR results confirmed the decreased expression of OmpK36 in the *kbvR* mutant. KbvR is a newly found regulator whose conserved DNA-binding motif is unknown. To determine whether KbvR directly triggers the activation of OmpK36, we mapped the genome-wide transcription-factor-binding sites of KbvR regulator by using DNA affinity purification sequencing (DAP-seq; Zoonbio biotechnology, Nanjing, China). DAP-seq is a transcription factor (TF)-binding site (TFBS) discovery assay that couples affinity-purified TFs with next-generation sequencing of a genomic DNA library (33).

**FIG 4:**
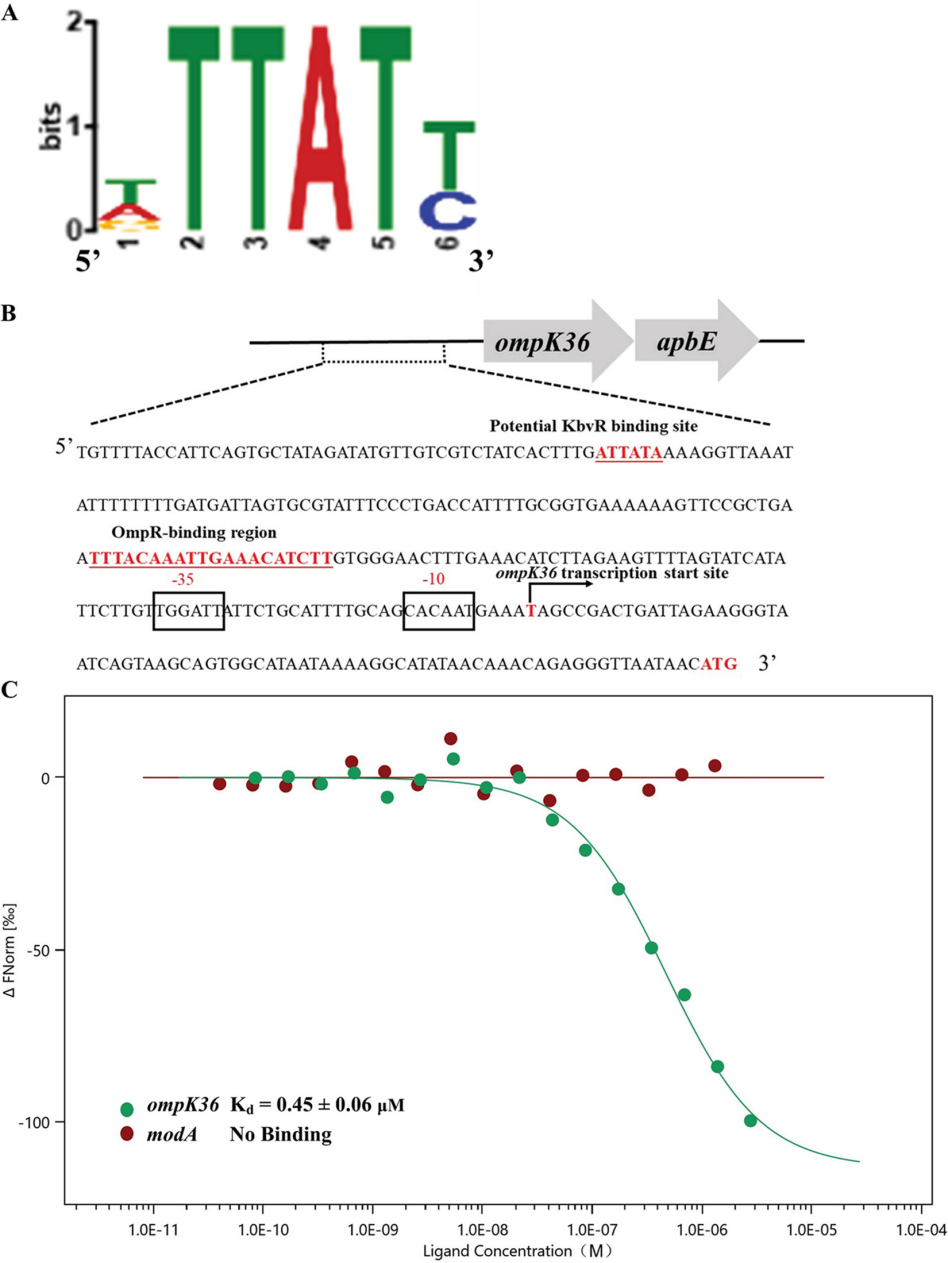
KbvR directly regulates the expression of OmpK36. (A) Conserved motif sequence of KbvR-binding sites specifically detected using DAP-seq followed by MEME analysis. (B) The *ompK36* gene and the promoter sequence. The large arrows represent the open-reading frames, and the potential KbvR- and OmpR-binding sites are marked in red and underlined. The initiation codon (ATG) is marked in red. The *ompK36* transcription start site is indicated by the arrow. The −35 region (TTGGAT) and −10 region (CACAAT) of the *ompK36* promoter are boxed. (C) Binding affinity of *ompK36* promoter region (green) and *modA* promoter region (red) to KbvR protein were determined using microscale thermophoresis (MST) analysis. The *K_d_* (dissociation constant) of *ompK36* is about 0.45 ± 0.06 μM, and *modA* has no *K_d_*.

On the basis of the genome, eight conserved motifs that might bind to KbvR were mapped by DAP-seq (data not shown). One putative binding motif was found at −253 to −248 bp relative to the start codon of *ompK36* (Fig. 4A and B). To validate the direct binding between KbvR and the promoter region of *ompK36*, the KbvR protein was expressed in E. coli and purified followed by fluorescent labeling. MST assay was performed with the labeled KbvR protein and different concentrations of a 186-bp PCR fragment containing the putative KbvR-binding motif in the *ompK36* promoter region. MST is a highly sensitive method for the analysis of the interactions between biomolecules, and equilibrium dissociation constant (*K_d_*) is used to show the affinity between a ligand and a receptor (34, 35). The result showed that the promoter region of *ompK36* caused concentration-dependent effects on the fluorescently labeled KbvR with a *K_d_* value of 0.45 ± 0.06 μM (Fig. 4C), indicating the specific binding of KbvR and the *ompK36* promoter region. The negative control of the promoter region of *modA* gene had no concentration-dependent effects and showed no binding affinity (no *K_d_* value). Therefore, KbvR can directly regulate the expression of OmpK36 to influence the outer membrane permeability and antibiotic resistance.

### Osmolarity decreases sensitivity to antibiotics through the regulation on OmpK36 by KbvR.

Environmental stresses frequently lead to increased sensitivity to other conditions. Exposure to environmental conditions, such as heavy metals or acidic or osmotic stress, may lead to increased antibiotic susceptibility (13, 14). The survival rates of WT and Δ*kbvR* strains against β-lactams (cefazolin and cefoxitin) were evaluated in physiological osmotic stress and high osmotic stress with 1% and 2.5% NaCl added in LB medium, respectively, to assess the influence of environmental osmotic stress on the antibiotic resistance of K. pneumoniae.

**FIG 5:**
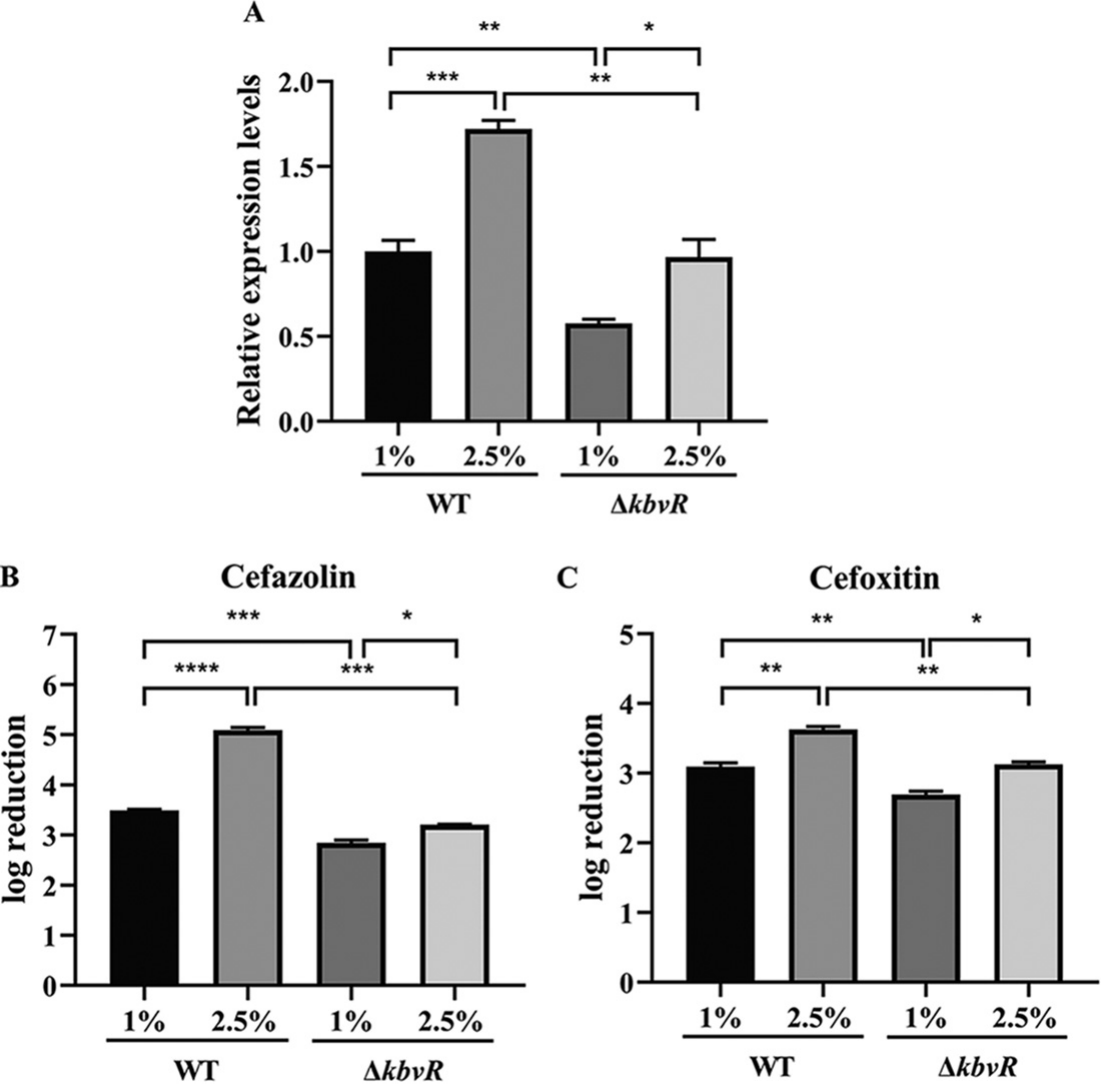
Osmolarity decreases antimicrobial resistance through the regulation on OmpK36 by KbvR. (A) mRNA levels of *ompK36* were determined by qRT-PCR in WT and Δ*kbvR* strains cultured in LB broth with 1% or 2.5% NaCl for 3 h. The transcript levels of the *ompK36* gene were determined relative to that of WT cultured in LB broth with 1% NaCl. (B and C) Influence of osmolarity on cefazolin and cefoxitin susceptibility. The WT and Δ*kbvR* strains were cultured in LB broth with 1% or 2.5% NaCl and then treated with 1,600 μg mL^−1^ cefazolin (B) or 800 μg mL^−1^ cefoxitin (C). The reduction of viable cells was determined by dividing the number of CFU mL^−1^ of the samples before antibiotic addition by the number of CFU mL^−1^ after 6 h of exposure to different antibiotics and converted logarithmically. Data are presented as mean ± SEM and representative of three independent experiments. *, *P < *0.05; **, *P < *0.01; ***, *P < *0.001; ****, *P < *0.0001 by Student’s *t* test.

Outer membrane proteins (OMPs) play a crucial role in adaptation to changes in surroundings (36). The transcription levels of outer membrane porin OmpK36 in WT and *ΔkbvR* exposed to high osmotic stress (2.5% NaCl) or physiological osmotic stress (1% NaCl) were determined by qRT-PCR. The fold induction of the OmpK36 gene was calculated by dividing transcription levels under different conditions by the OmpK36 transcription level of the WT strain grown in physiological osmotic stress (1% NaCl). The results showed increased OmpK36 expression in the WT strain under high osmotic stress (*P < *0.001) (Fig. 5A). Although the expression of OmpK36 in the Δ*kbvR* strain was higher under high osmotic stress than under physiological osmotic stress, the expression level was lower than that in the WT strain. These results indicated that KbvR is a factor but not the only factor in response to the change in osmotic stress to regulate OmpK36 expression; other factors such as OmpR are also involved in this process. The expression of OmpR, a well-known regulator associated with osmolarity, was increased in K. pneumoniae grown in LB medium supplemented with 2.5% NaCl (Fig. 1B).

The amount of OmpK36 outer membrane porin plays important roles in the development of β-lactam resistance (37). Porins confer β-lactams with the ability to overcome the hydrophobic barrier and enter the periplasm (38). In the WT strain, the high osmotic stress increased the expression of OmpK36 and improved the sensitivity to cefazolin and cefoxitin compared to that in the physiological osmotic stress. The viable cells were reduced by 1.24E + 05 versus 3.11E + 03 and 4.24E + 03 versus 1.25E + 03 at 2.5% and 1% NaCl concentrations for cefazolin and cefoxitin, respectively. When KbvR was deleted, the susceptibilities to cefazolin and cefoxitin in physiological and high osmotic stress were both decreased compared to those of WT. Meanwhile, the higher survival rate of the Δ*kbvR* mutant compared to that of the WT strain was even higher under high osmotic stress than under normal osmotic stress. The reduction folds of WT/Δ*kbvR* were 77.5 versus 4.4 and 3.2 versus 2.5 at 2.5% and 1% NaCl concentration for cefazolin and cefoxitin, respectively (Fig. 5B and C). The results suggested that the regulation of KbvR on the expression of OmpK36 had a role in the influence of osmolarity on antimicrobial sensitivity.

### Conclusions.

**FIG 6:**
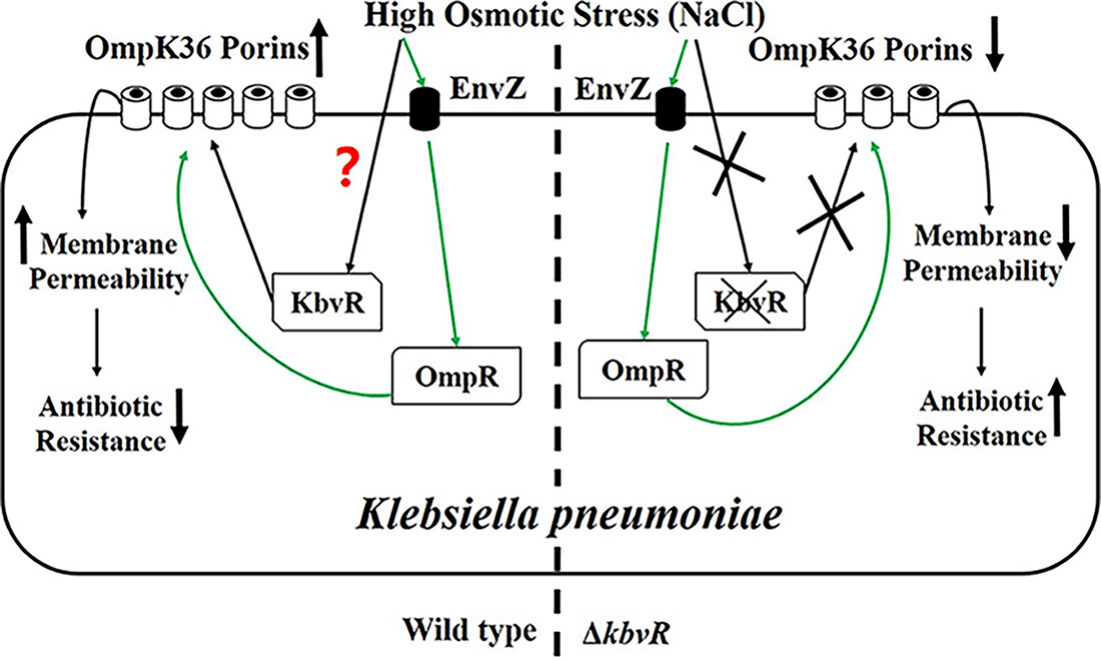
Graphical representation of osmotic stress increasing OmpK36 porin expression through the regulation of KbvR to decrease the antimicrobial resistance of K. pneumoniae. Green lines represent the pathway of the EnvZ/OmpR two-component system, a well-known regulator which can sense the change of osmotic stress and influence the expression of *ompK36* (16, 21). Black lines represent the pathway of KbvR which senses the high osmotic stress to increase the expression of OmpK36 with increased membrane permeability and decreased antibiotic resistance in this study. The question mark represents the unknown mechanism of whether KbvR senses the osmotic stress directly or is regulated by another primary sensor.

The sensing and adaption abilities of bacteria to the environmental osmotic stress can change the expression of their outer membrane porins, membrane permeability, and resistance to antibiotics. KbvR is a newly found regulator that can be upregulated under high osmotic stress and directly regulate the expression of OmpK36 to change the resistance of K. pneumoniae to β-lactam antibiotics (Fig. 6). In the wild-type strain, high osmotic stress is sensed by EnvZ, the sensor kinase of EnvZ/OmpR two-component signal transduction system. The activity of OmpR regulator is improved to increase the expression of OmpK36. Meanwhile, the stress stimulates the expression of OmpK36 by the regulation of KbvR regulator, whose mechanism to sense the osmotic stress is still unknown. The upregulated OmpK36 increases the membrane permeability, thereby facilitating the uptake of antibiotics and the access to their targets. In the *kbvR* deletion mutant strain, the high osmotic stress increases the expression of OmpK36 through regulation of OmpR but not KbvR, which ultimately upregulates the OmpK36 expression with increasing level lower than that of WT. The antimicrobial resistance in the Δ*kbvR* strain increases under high osmotic stress. The results demonstrate how adaptation to high osmotic stress changes the sensitivity of K. pneumoniae to antibiotics. The mechanism can be used to sensitize bacteria to antibiotics and highlight new potential strategies for exploiting shared constraints in governing adaptation to diverse environmental challenges.

## MATERIALS AND METHODS

### Bacterial strains and growth conditions.

Bacterial strains, plasmids, and primers used in this study are listed in Tables S1 and S2. The *kbvR* gene deletion strain Δ*kbvR* of K. pneumoniae NTUH-K2044 and the complementary strain C-*kbvR* were previously constructed in our laboratory (22). A PCR-generated DNA fragment containing the OmpK36-coding region together with its promoter-proximal region (540 bp upstream of the coding sequence) and putative transcriptional terminator (621 bp downstream) was cloned into the vector km-pGEM-T-easy to observe the role of OmpK36. The resulting plasmid was transformed into the *kbvR* mutant to obtain Δ*kbvR + ompK36* with upregulated expression of OmpK36. For all strains, the bacteria were grown overnight with shaking in LB broth with 1% NaCl (physiological osmotic stress) at 37°C. The cultures were adjusted to an optical density at 600 nm (OD_600_) of 1.2 and diluted 100-fold into fresh LB medium with different concentrations of sodium chloride (NaCl) or antibiotics as needed.

### Quantitative real-time PCR for analysis of gene expression.

The overnight cultures of strains were diluted 100-fold in fresh LB broth and incubated at 37°C to an OD_600_ of 1.6. Bacteria at 10^7^ CFU mL^−1^ were cultured in 3 mL of LB broth with 1% (physiological osmotic stress) or 2.5% NaCl (high osmotic stress) at 37°C for 3 h. Total RNA was isolated using an RNeasy mini column (Qiagen) according to the manufacturer’s recommendations. The RNA was treated with DNase (Promega) at 37°C for 30 min to eliminate chromosomal DNA. The reaction was terminated by adding the DNase stop solution at 65°C for 10 min. The extracted RNAs were reverse transcribed into cDNAs with oligonucleotide primers by using the first-strand cDNA synthesis kit (Promega). Quantitative real-time PCR (qRT-PCR) analysis was performed with the SYBR green supermix (Bio-Rad) in a light cycler CFX96 instrument, and 16S rRNA was used to normalize gene expression (39, 40).

### SDS-PAGE for analysis of protein OmpK36 expression.

SDS-PAGE was performed as described by Tsai et al. (17). The overnight cultures of WT, Δ*kbvR*, and Δ*kbvR + ompK36* strains were diluted by 1:100 into 3 mL fresh LB broth, respectively, and grown to an OD_600_ of 1.6. Then, the bacteria were collected at 12,000 rpm for 10 min, washed twice, resuspended with phosphate-buffered saline (PBS), and lysed by sonication. The concentration of protein was quantified by bicinchoninic acid (BCA) assay and normalized. The protein profiles were analyzed by SDS-PAGE using 12% SDS gels, followed by Coomassie blue staining. The relative intensity of protein OmpK36 band was quantitated by densitometry using ImageJ software. It was calculated by dividing the densitometry of OmpK36 band by the corresponding densitometry of total protein with the protein level of OmpK36 in WT defined as 1.

### Osmolarity assay.

The bacteria were precultured as described above. The bacteria were then cultured in 3 mL of fresh LB broth containing 1% (0.17 M), 1.5% (0.245 M), 1.9% (0.32 M), 2.5% (0.42 M), 3.2% (0.547 M), or 4% (0.67 M) NaCl at 10^7^ CFU mL^−1^ with shaking at 37°C for 3 h. The changes in salinity (NaCl) concentrations are assumed to correlate with changes in osmotic stress. The bacteria were serially diluted and plated on LB agar plates to calculate CFU. The survival rate was calculated by dividing the CFU of the samples treated with different concentrations of NaCl by the corresponding CFU of the samples with 1% NaCl in LB medium and multiplying by 100.

### Antibiotic susceptibility testing.

Antimicrobial susceptibility was determined using microdilution assays (41). The MICs of various antimicrobial agents were measured in 96-well microtiter plates. The overnight cultures of WT, Δ*kbvR*, C-*kbvR*, or Δ*kbvR *+ *ompK36* strains were diluted 100-fold in LB broth, incubated at 37°C to an OD_600_ of 1.2, and inoculated at a density of 5 × 10^5^ cells per mL into wells containing LB medium in the presence of 2-fold increased concentrations of antimicrobial agents. Cell growth was determined visually after incubation of the microtiter plates at 37°C for 18 h. MIC was defined as the lowest concentration of the antimicrobial agent that prevents the visible growth of a microorganism under defined antibiotics. All of the data were obtained from at least three independent experiments with at least three replicates.

The overnight cultures of strains WT and Δ*kbvR* were diluted 100-fold into LB broth with 1% or 2.5% NaCl and incubated for 3 h at 37°C to determine the influence of osmolarity on antibiotic susceptibility. Cells were collected and resuspended in LB broth with 1% NaCl. Bacteria at a density of 1 × 10^8^ cells mL^−1^ were exposed to cefazolin at 1,600 μg mL^−1^ and cefoxitin at 800 μg mL^−1^ (50 times the MIC for the Δ*kbvR* mutant) for 6 h at 37°C (42). The reduction of viable cells was determined by dividing the CFU mL^−1^ of the samples before antibiotic addition by the CFU mL^−1^ after 6 h of exposure to different antibiotics and converted logarithmically.

### Membrane permeability assay.

Membrane permeability assay was performed by measuring the permeabilization of the outer membrane to N-phenyl-1-naphthylamine (NPN; Aladdin), as described by Jung et al. (43). The overnight cultures were diluted by 1:100 into 3 mL of LB broth and grown to an OD_600_ of 1.6. A fresh culture of bacteria was centrifuged at 12,000 rpm for 10 min, washed twice with 5 mM HEPES buffer (pH 7.2), and suspended with 5 mM HEPES buffer. After 10 min of incubation with 10 μM carbonyl cyanide 3-chlorophenylhydrazone (CCCP; Macklin), 100 μL of the cell suspension was mixed with 100 μL of 10 μM NPN. Fluorescence signal (excitation, 355/15 nm; emission, 405/15 nm) was measured within 2 min on the SpectraMax i3 with record every 1 min for 30 min. All measurements were performed at room temperature.

### MST assay.

Microscale thermophoresis (MST) assay was performed as described by Jerabek–Willemsen et al. to validate the binding of KbvR protein and the promoter region of *ompK36* gene (44). Sixteen samples with constant concentration of fluorescently labeled KbvR protein and 2-fold increased concentrations of nonlabeled *ompK36* promoter probe including the putative binding motif of KbvR were mixed and incubated for 30 min at room temperature. Then, the specimens were loaded and measured using a Monolith NT.115 instrument (Nano Temper Technologies GmbH, Munich, Germany) at 30°C with 80% excitation power and medium LED power. The temperature-induced changes in fluorescence (temperature-related intensity changes and/or temperature-dependent movements) were determined as a function of *ompK36* target probe concentration in glass capillaries. Dissociation constant (*K_d_*) was calculated and fitted by Nano Temper Analysis software. Three independent measurements were analyzed using the signal from thermophoresis plus T-Jump. The promoter region of *modA*, a gene of K. pneumoniae which has no putative KbvR-binding motif in its promoter, was amplified and used as the negative-control ligand (45).

### Statistical analyses.

For all experiments, two-tailed Student’s *t* test and two-way analysis of variance (ANOVA) were performed to determine statistical significance. A difference in means was considered statistically significant if the *P* value was <0.05.
