# Supplementary material for: High Osmotic Stress Increases OmpK36 Expression through the Regulation of KbvR to Decrease the Antimicrobial Resistance of Klebsiella pneumoniae
Source: Microbiol Spectr. 2022 Jun 6;10(3):e00507-22. doi: 10.1128/spectrum.00507-22 (PMC9241633; doi:10.1128/spectrum.00507-22)
Supplement: Supplemental file 1 — Supplemental material. Download spectrum.00507-22-s0001.pdf, PDF file, 0.1 MB [file spectrum.00507-22-s0001.pdf]

Supplementary Table 1 Bacterial strains and plasmids used in this study

Strains and plasmids	Description	Reference
**Strains**		
Escherichia coli DH5α	F-φ80d/lacZΔM15 Δ(*lacZYA-argF*) U169 *deoR recA*1 *hsdR*17(rk- mk-) *phoA supE*44 λ-*thi*-1 *gyrA*96 *relA*1	Our lab
K. pneumoniae		
NTUH-K2044 (WT)	A capsular serotype K1 strain with hypermucoviscosity phenotype, wild-type strain; Ap^r	Our lab
Δ*kbvR*	Deletion of *kbvR* from WT; Ap^r	[1]
C-*kbvR*	Complemented *kbvR* mutant; Ap^r ; Km^r	[1]
Δ*kbvR*+*ompK36*	Deletion of *kbvR* strain with pGEM-*ompK36*; Ap^r ; Km^r	This study
**Plasmids**		
pKO3-Km	pKO3-derived plasmid, with an insertion of Km resistance cassette from pUC4K into *AccI* site	Our lab
pGEM-T-easy-km	pGEM-T easy with an insert of Km cassette from pUC4K into *NdeI* site for trans complementation	Our lab
pGEM-*ompK36*	pGEM-T-easy-derived vector containing the promoter, ORF and terminator region of *ompK36*	This study

Supplementary Table 2 Primers used in this study

Target gene	Primer sequences (forward/reverse, 5'-3')	Purpose
		**Construction of up-expression strain**
ompK36	GTATGCGGCCGCCACGAAATAACAAGACATT/<br>GTATGCGGCCGCCAAACAGATCGGGAATATCT	Amplification of 2279 bp fragment including the *ompK36* coding region with its promoter-proximal region (540 bp upstream of the coding sequence) and putative transcriptional terminator (621 bp downstream)
		**Detection by RT-PCR**
kbvR	GGACAATGAACACGCTACCG/CGCCGCAGACGATTGAAC	Amplification of a 165 bp intragenic region of *kbvR*
ompK36	CAACCTACCGTAACTCTGAT/GATGCCATCCCAAATATCGT	Amplification of a 179 bp intragenic region of *ompK36*
ompR	GAGCGTTATCTGACCGAGC/AGAGAGACCATCTTCGCCC	Amplification of a 135 bp intragenic region of *ompR*
**16S rRNA gene**	ATGACCAGCCACACTGGAAC/CTTCCTCCCCGCTGAAAGTG	Amplification of a 151 bp intragenic region of the 16sRNA gene
		**Detection by MST**
ompK36	GGTAAACAGACATTCAGAACTG/ATGGTCAGGGAAATACGCACTA	Amplification of the 186 bp DNA fragment upstream of the *ompK36*
modA	ACAGGCGCTGCTGTAGCTTA/AGAACCTGCCATCTTACTCT	Amplification of the DNA fragment upstream of the *modA*

Note: Underlined indicate restriction enzyme sites

REFERENCES

[1] Xu L, Wang M, Yuan J, et al. The KbvR regulator contributes to capsule production, outer membrane protein biosynthesis, antiphagocytosis, and virulence in *Klebsiella pneumoniae*. Infection and immunity. 2021;89(5).https://doi.org/10.1128/iai.00016-21
